# Parental burnout and borderline personality stand out to predict child maltreatment

**DOI:** 10.1038/s41598-023-39310-3

**Published:** 2023-07-27

**Authors:** Alice Schittek, Isabelle Roskam, Moïra Mikolajczak

**Affiliations:** UCLouvain, Place Cardinal Mercier 10, 1348 Louvain-la-Neuve, Belgium

**Keywords:** Psychology, Human behaviour

## Abstract

Parental burnout is a severe disorder resulting from the exposure to chronic stress in the parental role, that can translate into neglectful and violent parental behaviors towards the offspring. This study (N = 1003 parents) aims to examine the relative weight of parental burnout, job burnout, depression, generalized anxiety disorder, borderline personality, sadism, psychopathy, Machiavellianism, narcissism, and child abuse potential, in predicting violence and neglect towards the offspring. Social desirability was controlled. When all predictors are entered together in the model, violence *and* neglect towards the offspring are best predicted by borderline personality and parental burnout. Our results also indicate that sadism is a robust predictor of violence, however weaker than parental burnout and borderline personality. These results emphasize the importance of preventing parental burnout and supporting parents with borderline personality.

## Introduction

Parental burnout is a severe disorder resulting from the exposure to chronic stress in the parental role^1^ and affecting around 5–8% of parents. Parental burnout research recently boomed and unveiled the strong repercussion parental burnout has on parental behaviors towards the offspring. Parental burnout drastically increases neglectful and violent behaviors, and this has been observed in correlational^2^, cross-lagged^3^ and experimental^4^ designs.

However, violence and neglect (henceforth child maltreatment) are not only a consequence of parental burnout. Mood disorders and several personality traits also enhance the risk of child maltreatment^5,6^. As regards mood disorders, this is particularly the case for depression^7^ and stress/anxiety disorders^8^. As regards personality traits, the most documented risk factors are borderline personality^9^, sadism^10^, psychopathy^11^, Machiavellianism^12^ and narcissism^13^. Other child abuse risk factors (e.g., history of abuse, loneliness, …) have been gathered in the Child Abuse Potential^14^ which is logically also a strong predictor of child maltreatment.

While many psychological disorders or traits increase the risk of child maltreatment, no study has ever compared their relative weight in a single model. Whether parental burnout deserves specific attention is therefore unknown. Controlling for the effect of other predictors is all the more important as they are not independent from each other. The aim of this study is to examine the relative weight of parental burnout, job burnout, depression, generalized anxiety disorder, borderline personality, sadism, psychopathy, Machiavellianism, narcissism, and child abuse potential, in predicting violence and neglect towards the offspring. We expected that parental burnout, job burnout, depression, generalized anxiety disorder, borderline personality, sadism, psychopathy, Machiavellianism, narcissism, and child abuse potential, would be positively correlated with violence and neglect towards the offspring. As regards to relative weight of each of the aforementioned variables when entered together in the same model, we did not have specific hypotheses, although some variables (e.g., parental burnout, borderline personality, and child abuse potential) were more expected to stand out because their bivariate correlations with child maltreatment in previous studies were of large magnitude. Considering that violence towards the offspring is a taboo subject in current society, it is subject to social desirability. Thus, we included it as a variable in both regression models, allowing to interpret results whilst controlling for social desirability.

## Method

### Procedure

The study was designed and carried out in accordance with relevant guidelines and regulations, such as the Declaration of Helsinki, and it received approval from the ethical committee of Cliniques Universitaires Saint-Luc (Belgium). Participants had to be parents and had to have at least one child still living at home. The study was created on Qualtrics, and it was posted on various online groups, websites, social media networks. Participants gave their free and informed consent prior to participating in the study. As an additional motivation, after participating parents could sign up to take part to a raffle by leaving their e-mail address (automatically disconnected from their questionnaire to ensure anonymity), with the possibility of winning 250 euros. Statistical analyses were conducted using SPSS, version 28^15^.

### Participants

In total, 1494 parents participated to the study. Given our aim to compare predictors’ relative weights, only participants who completed the entire survey were kept (N = 680; 88.5% mothers). Survey participants were recruited through social media, websites, youth movement groups and word of mouth. Analyses were carried out to determine whether values were missing due to random reasons, or due to specific reasons. T-tests revealed that the values where not missing at random, in fact missing participants tended to be more women (*t*(998) = −2.9, *p* = 0.003), slightly less well educated (*t*(998) = 2.28, *p* = 0.005), mostly not working (*t*(998) = −23.4, *p* < 0.001), slightly poorer (*t*(998) = 5.28, *p* < 0.001), and with a higher level of parental burnout (*t*(998) = −25.1, *p* < 0.001). The authors would also like to point out that missingness was proportionate with the length of the study (i.e., we observed a consistent decrease in responses as the questionnaires went on). However, although we observed a consistent decrease in responses for most of the study, there was a sudden drop of participation when the participants were faced with the sadism measure, showing how uncomfortable it can be when answering that questionnaire. For ethical reasons, we did not force the respondents to answer all the questions, thus leaving us with 680 participants. In the current sample, the average age was 38.6 years. Most parents had completed higher education, such as bachelor’s degree (41.8%%) and master’s degree (29.3%), followed by higher secondary education (17.4%), third cycle education (5.9%), lower secondary education (4.7%), and primary education (1%). Almost half of the sample (41.0%) had a monthly income of 2500–4000 Euros, followed by 4000–5500 Euros (27.4%), 1000–2500 Euros (17.5%), 5500–7000 Euros (10.9%), higher than 7000 Euros (2.6%), and 0–1000 Euros (0.6%). Most parents were either married (47.6%) or legally cohabitating (35.9%), and 16.5% were in single-parenting situations. The majority of parents lived in Belgium (82.1%) or France (16.2%). About 89.3% of the sample had between 1 and 3 children, and for more than half of the parents (53.1%) the first child was between 1 and 12 years of age (*Mean* = 11 years; *Standard Deviation* = 8.19). Thus, compared to the population of Belgium and France, the current sample was over-representative of mothers and not fathers, slightly over-educated, just-representative in terms of income, representative in terms of married and legal-cohabitating parents, slightly over-representative of single-parenting situations, and representative of the populations in terms of number of children.

### Measures

Parental burnout was measured using the Parental Burnout Assessment (PBA; α = 0.97;^16^), which encompasses 23 items (e.g., *I’m so tired out by my role as a parent that sleeping doesn’t seem like enough*) rated on a 7-point frequency scale: Never (0), a few times a year (1), once a month or less (2), a few times a month (3), once a week (4), a few times a week (5), every day (6). Total score was computed by summing item responses together, and for all measures hereafters, except if stated otherwise. Job burnout was assessed using the Maslach Burnout Inventory General Survey^17^, which encompasses 16 items rated (e.g., *I feel used up at the end of the work day*) on the same 7-point Likert frequency scale as above (α = 0.88). Depression was measured with the Patient Health Questionnaire^18^, including 9 items (e.g., *little interest or pleasure in doing things*) representing each DSM symptom of depression. Participants indicated how often they have experienced specific symptoms during the last two weeks on a frequency scale: Not at all (0), Several Days (1), More than Half the Days (2), Nearly Every Day (3) (α = 0.89). Generalized Anxiety was assessed with the GAD-7^19^, a 7-item questionnaire (e.g., *feeling nervous, anxious or on edge*) measuring the presence of anxiety symptoms during the last 14 days on a 4-point frequency scale: Not at all (0), Several days (1), More than half the days (2), and nearly every day (3) (α = 0.92). Borderline Personality was assessed with the Borderline Evaluation of Severity over Time (BEST;^20^), which is a 15-item (e.g., *going to extremes to try to keep someone from leaving you*) questionnaire (α = 0.86). For the first 12 items, the Likert scale goes from “None/Slight” (1) to “Extreme” (5), whereas for items 13–15, the scores go from “Almost Never” (1) to “Almost Always” (5). Sadism was measured using the Assessment of Sadistic Personality (ASP;^21^), which is a 9-item questionnaire (e.g., *I think about hurting people who irritate me*) rated on a 5-point Likert scale from 1 (strongly disagree) to 5 (strongly agree) (α = 0.82). Psychopathy, Machiavellianism, and narcissism were measured using the Short Dark Triad Scale^22^, a 27-item questionnaire (e.g., *I like to get revenge on authorities*) in which each aforementioned variable is measured with 9 items rated on a 5-point Likert scale from “Disagree strongly” (1) to “Agree strongly” (5) (Machiavellianism α = 0.78, narcissism α = 0.64, psychopathy α = 0.71). Total score for the three subscales were created by averaging the items. Child Abuse Potential was assessed using the Brief Child Abuse Potential (BCAP;^23,24^), a questionnaire encompassing 21 items (e.g., *my family has problems getting along*) rated on a binary scale: agree (0) or disagree (1) (α = 0.86). Total score was computed by averaging the items. Violence and neglect were measured using the Neglect and Violence Scales^2^. The former encompasses 17 items (e.g., *I sometimes don’t react when my child tells me something*) , the latter 15 items (e.g., *I sometimes spank or slap my child*), all rated on a 8-point frequency scale: never (0), less than once a month (1), about once a month (2), a few times a month (3), once a week (4), several times a week (5), every day (6), several times a day (7) (for violence α = 0.80, for neglect α = 0.72). Total scores were created by averaging the items. Social desirability was measured using the short form of the Marlowe-Crowne Social Desirability scale^25^, which is a 12-item (e.g., *I sometimes feel resentful when I don’t get my way*) questionnaire rated on a binary scale: true (1) or false (0) (α = 0.60).

### Statistical analyses

Analyses were computed with list-wise deletion, thus only keeping participants who responded to all the items. In order to test our hypotheses, we calculated bivariate correlations between variables, and then we computed hierarchical regression analyses, by firstly introducing the control variable (i.e., social desirability) and secondly the remaining variables. Hierarchical regression models were tested with and without a bootstrapping of 1000 samples to ensure that presented results are robust.

## Results

Correlation estimates and their significance level can be seen in Table 1. As expected, with the exception of Narcissism, all variables were positively and significantly correlated to both neglect and violence.

**Table 1:** Bivariate intercorrelations between variables.

Variable	*n*	*M*	*SD*	1	2	3	4	5	6	7	8	9	10	11	12	13
1. MBI	680	32.14	16.60	–												
2. PBA	680	24.05	23.47	0.36**	–											
3. PHQ-9	680	6.83	5.11	0.45**	0.62**	–										
4. GAD-7	680	5.98	4.78	0.38**	0.53**	0.78**	–									
5. Narcissism	680	2.72	0.49	−0.13**	−0.03	−0.07	−0.09*	–								
6. Psychopathy	680	1.89	0.53	0.12**	0.08*	0.14**	0.16**	0.17**	–							
7.Machiavellianism	680	2.64	0.61	0.11**	0.07	0.15**	0.16**	0.14**	0.47**	–						
8. ASP	680	1.69	0.46	0.05	0.11**	0.11**	0.10*	0.16**	0.41**	0.33**	–					
9. BCAP	680	1.31	0.22	0.36**	0.52**	0.68**	0.62**	−0.06	0.24**	0.24**	0.11**	–				
10. BEST	680	25.13	8.66	0.39**	0.61**	0.70**	0.64**	−0.04	0.27**	0.22**	0.18**	0.65**	–			
11. Neglect	680	1.62	0.49	0.24**	0.43**	0.33**	0.26**	−0.06	0.12**	0.11**	0.12**	0.25**	0.48**	–		
12. Violence	680	1.45	0.45	0.17**	0.54**	0.37**	0.34**	−0.02	0.21**	0.15**	0.20**	0.36**	0.56**	0.53**	–	
13. Desirability	680	18.14	2.39	−0.21**	−0.14**	−0.17**	−0.17**	−0.04	−0.39**	−0.33**	−0.27**	−0.23**	−0.29**	−0.19**	−0.18**	–

Desirability = Social Desirability; MBI = Maslach Burnout Inventory General Survey; PBA = Parental Burnout Assessment; PHQ-9 = Patient Health Questionnaire; GAD-7 = Generalized Anxiety Disorder; ASP = Assessment of Sadistic Personality; BCAP = Brief Child Abuse Potential; BEST = Borderline Evaluation of Severity over Time.

*p < 0.05. **p < 0.01.

Hierarchical regression analyses (see Tables 2 and 3) were used to examine the relative weight of the various predictors of violence and of neglect while controlling for social desirability. We decided to leave narcissism in the model because the absence of bivariate correlation could be due to high social desirability in narcissistic individuals. When predicting violence, the global model was significant (*F*(11, 668) = 41.01, *p* < 0.001; R^2^ = 0.40). In this model, only four predictors remained significant: Parental burnout (*t*(668) = 9.15, *p* < 0.001) and borderline personality (*t*(668) = 9, p < 0.001) stood out as strong predictors of violence; sadism also stood out but to a lesser extent (*t*(668) = 2.1, *p* = 0.04). Job burnout (*t*(668) = −2.06, *p* = 0.04) was also significant, but it *negatively* predicted violence (thus, compared to bivariate correlations, its sign reversed). The model remained the same even after bootstrapping of 1000 samples.

**Table 2:** Hierarchical regression analysis: Variables predicting violence, with desirability controlled.

Effect	Standardized Estimates	SE	F	t	*p*	R square
Model predicting violence			41.01		< 0.001**	0.40
Desirability	0.002	0.01		0.052	0.96	
MBI	−0.07	0.001		−2.06	0.04*	
PBA	0.37	0.001		9.15	< 0.001**	
PHQ-9	−0.10	0.01		−1.82	0.07	
GAD-7	−0.03	0.01		−0.50	0.62	
Narcissism	−0.004	0.03		0.12	0.91	
Psychopathy	0.06	0.03		1.70	0.09	
Machiavellianism	0.01	0.03		0.23	0.82	
ASP	0.07	0.03		2.1	0.04*	
BCAP	−0.03	0.09		−0.75	0.45	
BEST	0.44	0.003		9.0	< 0.001**	

N = 680, excluding cases listwise. Desirability = Social Desirability; MBI = Maslach Burnout Inventory General Survey; PBA = Parental Burnout Assessment; PHQ-9 = Patient Health Questionnaire; GAD-7 = Generalized Anxiety Disorder; ASP = Assessment of Sadistic Personality; BCAP = Brief Child Abuse Potential; BEST = Borderline Evaluation of Severity over Time. Bootstrap of 1000 samples.

**Table 3:** Hierarchical regression analysis: Variables predicting neglect, with desirability controlled.

Effect	Standardized estimates	SE	F	t	*p*	R square
Model predicting neglect			24.38		< 0.001**	0.29
Desirability	−0.06	0.01		−1.60	0.11	
MBI	0.05	0.001		1.3	0.19	
PBA	0.26	0.001		5.9	< 0.001**	
PHQ-9	0.01	0.01		0.14	0.89	
GAD-7	−0.10	0.01		−1.9	0.06	
Narcissism	−0.05	0.03		−1.4	0.16	
Psychopathy	−0.003	0.04		−0.08	0.94	
Machiavellianism	0.02	0.03		0.53	0.57	
ASP	0.02	0.04		0.62	0.53	
BCAP	−0.14	0.11		−2.9	0.004**	
BEST	0.42	0.003		7.9	< 0.001**	

N = 680, excluding cases listwise*.* Desirability = Social Desirability; MBI = Maslach Burnout Inventory General Survey; PBA = Parental Burnout Assessment; PHQ-9 = Patient Health Questionnaire; GAD-7 = Generalized Anxiety Disorder; ASP = Assessment of Sadistic Personality; BCAP = Brief Child Abuse Potential; BEST = Borderline Evaluation of Severity over Time. Bootstrap of 1000 samples.

Concerning neglect, the global model was significant (*F*(11,668) = 24.38, *p* < 0.001; R^2^ = 0.29). Among all the predictors, only borderline personality (*t*(11,668) = 7.9, *p* < 0.001) and parental burnout (*t*(11,668) = 5.9, *p* < 0.001) stood out as positive predictors of neglect. Child abuse potential also stood out but it was *negatively* related to neglect (*t*(11,668) = −2.9, *p* = 0.004) (thus, compared to bivariate correlations, its sign reversed). The model remained the same even after bootstrapping of 1000 samples.

## Discussion

The aim of the current study was to examine the relative weight of various predictors of child maltreatment. Our findings suggest that violence *and* neglect towards the offspring are best predicted by borderline personality and parental burnout. Both variables are consistently significant across both violence and neglect; they are robust with relatively high effect sizes, even when social desirability is controlled for. This corroborates previous findings that parental burnout has specific repercussions on children, and it also adds to prior literature concerning the implications of borderline personality. Because borderline personality and parental burnout are highly correlated, this suggests that the former may be a vulnerability factor for the latter. The fact that parental burnout predicts both neglect and violence even when borderline personality is controlled for, suggests that parental burnout has significant consequences on the offspring even in non-borderline parents. Finally, our results also corroborate previous research that sadism is a robust predictor of violence^26^. We will not dwell on the suppressor effects of job burnout and BCAP (i.e., reversion of the sign when we control for all the other predictors), as their bivariate correlations were positive.

The implications of these findings are consequential, both for research and clinical practice. To begin, the importance of parental burnout prevention is key, as it is a robust and consistent predictor of violence and neglect towards the offspring. Knowing that parental burnout develops in stages and that it begins with emotional exhaustion^27^ and knowing that it is especially when parents are emotionally distant from their children that neglect and violence kick in^28^, identifying parents at the exhaustion stage (i.e., before the stage of distancing) is crucial in order to treat the parents and, by doing so, preventing neglect or violence to occur. Also, the current study identified borderline personality as another factor of tremendous importance in predicting violence and neglect towards the children. Knowing this, prevention should be reinforced. Until 2015, few programs to support parenting in parents with borderline personality existed but things are starting to change^29,30^; the current findings suggests that these endeavors should be supported.

As advised by reviewers, additional analyses were performed. First, the previous regression models were re-analyzed by adding socio-demographic variables. In predicting neglect, results show that parental burnout, narcissism, abuse potential, being a man, and having younger children increases the risk of neglect. In predicting violence, parental burnout, borderline personality, and lower levels of education increase the risk of violence (results can be viewed in Supplementary Tables 1 and 2). Second, moderation analyses were conducted to see whether the observed effects varied based on socio-demographic variables. Analyses revealed that borderline personality disorder increases the prediction of neglect more so in men than in women. Also, Machiavellianism predicts more neglect in older parents compared to younger ones. Regarding violence towards the offspring, moderation analyses revealed that violence was predicted more by child abuse potential in older parents, and also by borderline personality disorder in younger parents. Age of children never showed a moderating effect in neither the prediction of neglect nor of violence. Moderation analyses can be viewed in Supplementary Tables (3–8). These results show that even when adding socio-demographic variables, parental burnout and borderline personality disorder still stand out in predicting child maltreatment.

Despite its strengths, this study also has limitations. The main one is that the study is correlational, precluding causal interpretations. However, the causal effect of parental burnout on violence and neglect towards the offspring has already been demonstrated^4^. Also, the authors did not account for the fact that some parents in the sample could be from the same family, this precluding the independence of observations. However, considering that the vast majority of participants were mothers, it is not very likely that this bias had an effect on the statistical analyses. Future research should be conducted to assess whether borderline personality is a precursor of parental burnout, whether/how borderline personality interacts with parental burnout, and whether the interaction has an additive effect on the violent and neglectful behavioral outbursts.

## Supplementary Information

Supplementary Tables.

## Data Availability

All data have been made publicly available via Open Science Framework and can be accessed at https://osf.io/47q25/?view_only=b6c6ea4ffd6b4b9486c5a467b2be72f0.
