# Supplementary material for: Parental burnout and borderline personality stand out to predict child maltreatment
Source: Sci Rep. 2023 Jul 27;13:12153. doi: 10.1038/s41598-023-39310-3 (PMC10374633; doi:10.1038/s41598-023-39310-3)
Supplement: Supplementary file 1 — Supplementary Tables. [file 41598_2023_39310_MOESM1_ESM.docx]

**Supplementary material**

**Supplementary material 1**

*Hierarchical regression analysis: Variables predicting neglect, with desirability controlled, and socio-demographic variables added*

| Effect | Standardized Estimates | SE | F | t | p | R square |
| --- | --- | --- | --- | --- | --- | --- |
|  |  |  |  |  |  |  |
| Model predicting neglect |  |  | 16.71 |  | < .001** | .31 |
| Desirability | -.08 | .01 |  | -2.13 | .03* |  |
| MBI | .07 | .001 |  | 1.95 | .05 |  |
| PBA | .29 | .001 |  | 6.28 | < .001** |  |
| PHQ-9 | -.02 | .01 |  | -.31 | .78 |  |
| GAD-7 | -.07 | .01 |  | -1.26 | .20 |  |
| Narcissism | -.08 | .04 |  | -2.17 | .04* |  |
| Psychopathy | -.02 | .04 |  | -.43 | .68 |  |
| Machiavellianism | .02 | .03 |  | -.37 | .74 |  |
| ASP | .03 | .04 |  | .69 | .43 |  |
| BCAP | -.13 | .12 |  | -2.67 | .02* |  |
| BEST | .43 | .01 |  | 8.14 | < .001** |  |
| Gender | -.09 | .06 |  | -2.40 | .02* |  |
| Age | -.05 | .01 |  | -.91 | .57 |  |
| Child’s Age | .19 | .01 |  | 3.26 | .02* |  |
| Marital Status | .001 | .03 |  | .03 | .98 |  |
| Education Level | .04 | .02 |  | .97 | .33 |  |
| Work Regimen | .02 | .02 |  | .52 | .58 |  |
| Income | .07 | .02 |  | 1.90 | .07 |  |

*Note.* N = 680, excluding cases listwise*.* Desirability = Social Desirability; MBI = Maslach Burnout Inventory General Survey; PBA = Parental Burnout Assessment; PHQ-9 = Patient Health Questionnaire; GAD-7 = Generalized Anxiety Disorder; ASP = Assessment of Sadistic Personality; BCAP = Brief Child Abuse Potential; BEST = Borderline Evaluation of Severity over Time. Bootstrap of 1000 samples.

**Supplementary material 2**

*Hierarchical regression analysis: Variables predicting violence, with desirability controlled, and socio-demographic variables added*

| Effect | Standardized Estimates | SE | F | t | p | R square |
| --- | --- | --- | --- | --- | --- | --- |
|  |  |  |  |  |  |  |
| Model predicting violence |  |  | 27.49 |  | < .001** | .43 |
| Desirability | -.01 | .01 |  | -.36 | .71 |  |
| MBI | .05 | .001 |  | -1.53 | .18 |  |
| PBA | .38 | .001 |  | 9.29 | < .001** |  |
| PHQ-9 | -.08 | .01 |  | -1.41 | .22 |  |
| GAD-7 | -.03 | .004 |  | -.64 | .48 |  |
| Narcissism | .01 | .03 |  | -.34 | .73 |  |
| Psychopathy | .06 | .04 |  | 1.53 | .21 |  |
| Machiavellianism | -.002 | .03 |  | -.05 | .98 |  |
| ASP | .06 | .04 |  | 1.74 | .12 |  |
| BCAP | -.05 | .10 |  | -1.13 | .28 |  |
| BEST | .44 | .004 |  | 9.07 | < .001** |  |
| Gender | -.01 | .05 |  | -.19 | .86 |  |
| Age | -.13 | .01 |  | -2.48 | .31 |  |
| Child’s Age | .15 | .01 |  | 2.87 | .18 |  |
| Marital Status | -.03 | .02 |  | -.74 | .49 |  |
| Education Level | -.08 | .02 |  | -2.38 | .02* |  |
| Work Regimen | -.01 | .02 |  | -.34 | .21 |  |
| Income | .08 | .02 |  | 2.25 | .09 |  |

*Note.* N = 680, excluding cases listwise*.* Desirability = Social Desirability; MBI = Maslach Burnout Inventory General Survey; PBA = Parental Burnout Assessment; PHQ-9 = Patient Health Questionnaire; GAD-7 = Generalized Anxiety Disorder; ASP = Assessment of Sadistic Personality; BCAP = Brief Child Abuse Potential; BEST = Borderline Evaluation of Severity over Time. Bootstrap of 1000 samples.

**Supplementary material 3**

*Hierarchical regression analysis: Variables predicting neglect, with desirability controlled, and age of first child as a moderator*

| Effect | Standardized Estimates | SE | F | t | p | R square |
| --- | --- | --- | --- | --- | --- | --- |
|  |  |  |  |  |  |  |
| Model predicting neglect |  |  | 25.07 |  | < .001** | .33 |
| Desirability | -.07 | .01 |  | -1.8 | .06 |  |
| MBI | .07 | .002 |  | 1.1 | .27 |  |
| PBA | .19 | .002 |  | 2.4 | .05 |  |
| PHQ-9 | -.04 | .01 |  | -.39 | .74 |  |
| GAD-7 | -.06 | .01 |  | -.68 | .52 |  |
| Narcissism | -.01 | .05 |  | -.14 | .89 |  |
| Psychopathy | .04 | .07 |  | .53 | .59 |  |
| Machiavellianism | -.03 | .05 |  | -.38 | .70 |  |
| ASP | .01 | .04 |  | .20 | .81 |  |
| BCAP | -.21 | .06 |  | -3.0 | .004** |  |
| BEST | .36 | .16 |  | 4.07 | < .001** |  |
| MBI*Age Child | .001 | .01 |  | .01 | .98 |  |
| PBA*Age Child | .16 | .001 |  | 2.10 | .07 |  |
| PHQ-9*Age Child | .04 | .001 |  | .35 | .74 |  |
| GAD-7*Age Child | -.04 | .001 |  | -.43 | .64 |  |
| Narcissism*Age Child | -.24 | .004 |  | -1.29 | .18 |  |
| Psychopathy*Age Child | -.13 | .005 |  | -.82 | .42 |  |
| Machiavellianism*Age Child | .19 | .004 |  | 1.04 | .27 |  |
| ASP*Age Child | -.04 | .004 |  | -.28 | .77 |  |
| BCAP*Age Child | .07 | .01 |  | .31 | .76 |  |
| BEST*Age Child | .22 | .001 |  | 1.37 | .29 |  |

*Note.* N = 680, excluding cases listwise*.* Desirability = Social Desirability; MBI = Maslach Burnout Inventory General Survey; PBA = Parental Burnout Assessment; PHQ-9 = Patient Health Questionnaire; GAD-7 = Generalized Anxiety Disorder; ASP = Assessment of Sadistic Personality; BCAP = Brief Child Abuse Potential; BEST = Borderline Evaluation of Severity over Time. Variables that are “variable name*Age Child” consist of interaction term to test the moderating effect of the age of the first child. Bootstrap of 1000 samples.

**Supplementary material 4**

*Hierarchical regression analysis: Variables predicting violence, with desirability controlled and age of first child as a moderator*

| Effect | Standardized Estimates | SE | F | t | p | R square |
| --- | --- | --- | --- | --- | --- | --- |
|  |  |  |  |  |  |  |
| Model predicting violence |  |  | 22.59 |  | < .001** | .42 |
| Desirability | -.0.3 | .01 |  | -.09 | .91 |  |
| MBI | -.11 | .002 |  | -1.98 | .07 |  |
| PBA | .31 | .002 |  | 4.31 | .01* |  |
| PHQ-9 | .04 | .01 |  | .41 | .73 |  |
| GAD-7 | -.08 | .01 |  | -.92 | .34 |  |
| Narcissism | .05 | .05 |  | .90 | .39 |  |
| Psychopathy | .02 | .06 |  | .37 | .74 |  |
| Machiavellianism | .04 | .05 |  | .72 | .48 |  |
| ASP | .03 | .06 |  | .48 | .62 |  |
| BCAP | -.06 | .14 |  | -.98 | .34 |  |
| BEST | .42 | .01 |  | 5.02 | < .001** |  |
| MBI*Age Child | .08 | .001 |  | .94 | .40 |  |
| PBA*Age Child | .07 | .001 |  | 1.01 | .33 |  |
| PHQ-9*Age Child | -.18 | .001 |  | -1.70 | .10 |  |
| GAD-7*Age Child | .07 | .001 |  | .72 | .37 |  |
| Narcissism*Age Child | -.21 | .003 |  | -1.20 | .20 |  |
| Psychopathy*Age Child | .13 | .004 |  | .90 | .35 |  |
| Machiavellianism*Age Child | -.13 | .003 |  | -.78 | .41 |  |
| ASP*Age Child | .11 | .004 |  | .81 | .40 |  |
| BCAP*Age Child | .04 | .01 |  | .20 | .83 |  |
| BEST*Age Child | .11 | .001 |  | .69 | .62 |  |

*Note.* N = 680, excluding cases listwise*.* Desirability = Social Desirability; MBI = Maslach Burnout Inventory General Survey; PBA = Parental Burnout Assessment; PHQ-9 = Patient Health Questionnaire; GAD-7 = Generalized Anxiety Disorder; ASP = Assessment of Sadistic Personality; BCAP = Brief Child Abuse Potential; BEST = Borderline Evaluation of Severity over Time. Variables that are “variable name*Age Child” consist of interaction term to test the moderating effect of the age of the first child. Bootstrap of 1000 samples.

**Supplementary material 5**

*Hierarchical regression analysis: Variables predicting neglect, with desirability controlled, and gender of parent as a moderator*

| Effect | Standardized Estimates | SE | F | t | p | R square |
| --- | --- | --- | --- | --- | --- | --- |
|  |  |  |  |  |  |  |
| Model predicting neglect |  |  | 14.1 |  | < .001** | .31 |
| Desirability | -.07 | .01 |  | -1.7 | .09 |  |
| MBI | -.05 | .01 |  | -.18 | .85 |  |
| PBA | .09 | .01 |  | .25 | .81 |  |
| PHQ-9 | -.29 | .04 |  | -.86 | .42 |  |
| GAD-7 | .30 | .06 |  | .65 | .54 |  |
| Narcissism | .24 | .21 |  | 1.20 | .20 |  |
| Psychopathy | -.45 | .23 |  | -1.20 | .05 |  |
| Machiavellianism | .23 | .22 |  | 1.02 | .38 |  |
| ASP | -.01 | .27 |  | -.06 | .96 |  |
| BCAP | -.62 | .69 |  | -2.1 | .04* |  |
| BEST | 1.41 | .03 |  | 4.0 | < .001** |  |
| MBI*Gender Parent | .11 | .01 |  | .36 | .69 |  |
| PBA*Gender Parent | .21 | .004 |  | .60 | .56 |  |
| PHQ-9*Gender Parent | .28 | .02 |  | .81 | .45 |  |
| GAD-7*Gender Parent | -.39 | .03 |  | -.80 | .46 |  |
| Narcissism*Gender Parent | -.40 | .11 |  | -1.51 | .12 |  |
| Psychopathy*Gender Parent | .49 | .12 |  | 1.9 | .07 |  |
| Machiavellianism*Gender Parent | -.26 | .12 |  | -.94 | .42 |  |
| ASP*Gender Parent | .03 | .14 |  | .12 | .92 |  |
| BCAP*Gender Parent | .68 | .35 |  | 1.6 | .10 |  |
| BEST*Gender Parent | -1.13 | .02 |  | -2.8 | .03* |  |

*Note.* N = 680, excluding cases listwise*.* Desirability = Social Desirability; MBI = Maslach Burnout Inventory General Survey; PBA = Parental Burnout Assessment; PHQ-9 = Patient Health Questionnaire; GAD-7 = Generalized Anxiety Disorder; ASP = Assessment of Sadistic Personality; BCAP = Brief Child Abuse Potential; BEST = Borderline Evaluation of Severity over Time. Variables that are “variable name*Gender Parent” consist of interaction term to test the moderating effect of the gender of the parent. Bootstrap of 1000 samples.

**Supplementary material 6**

*Hierarchical regression analysis: Variables predicting violence, with desirability controlled, and gender of parent as a moderator*

| Effect | Standardized Estimates | SE | F | t | p | R square |
| --- | --- | --- | --- | --- | --- | --- |
|  |  |  |  |  |  |  |
| Model predicting violence |  |  | 22.95 |  | < .001** | .42 |
| Desirability | .01 | .01 |  | .38 | .73 |  |
| MBI | -.43 | .01 |  | -1.6 | .07 |  |
| PBA | .43 | .01 |  | 1.37 | .40 |  |
| PHQ-9 | -.36 | .05 |  | -1.19 | .57 |  |
| GAD-7 | .03 | .05 |  | .07 | .95 |  |
| Narcissism | .03 | .18 |  | .13 | .91 |  |
| Psychopathy | .06 | .21 |  | .29 | .79 |  |
| Machiavellianism | -.29 | .26 |  | -1.45 | .45 |  |
| ASP | .42 | .23 |  | 2.03 | .06 |  |
| BCAP | -.40 | .80 |  | -1.46 | .32 |  |
| BEST | 1.46 | .05 |  | 4.5 | .04* |  |
| MBI*Gender Parent | .38 | .003 |  | 1.40 | .13 |  |
| PBA*Gender Parent | -.04 | .01 |  | -.12 | .94 |  |
| PHQ-9*Gender Parent | .26 | .03 |  | .80 | .69 |  |
| GAD-7*Gender Parent | -.04 | .03 |  | -.09 | .95 |  |
| Narcissism*Gender Parent | -.03 | .09 |  | -.11 | .91 |  |
| Psychopathy*Gender Parent | -.001 | .11 |  | -.01 | .98 |  |
| Machiavellianism*Gender Parent | .39 | .13 |  | 1.52 | .41 |  |
| ASP*Gender Parent | -.41 | .12 |  | -1.76 | .11 |  |
| BCAP*Gender Parent | .53 | .41 |  | 1.37 | .37 |  |
| BEST*Gender Parent | -1.17 | .02 |  | -3.20 | .25 |  |

*Note.* N = 680, excluding cases listwise*.* Desirability = Social Desirability; MBI = Maslach Burnout Inventory General Survey; PBA = Parental Burnout Assessment; PHQ-9 = Patient Health Questionnaire; GAD-7 = Generalized Anxiety Disorder; ASP = Assessment of Sadistic Personality; BCAP = Brief Child Abuse Potential; BEST = Borderline Evaluation of Severity over Time. Variables that are “variable name*Gender Parent” consist of interaction term to test the moderating effect of the gender of the parent. Bootstrap of 1000 samples.

**Supplementary material 7**

*Hierarchical regression analysis: Variables predicting neglect, with desirability controlled, and age of parent as a moderator*

| Effect | Standardized Estimates | SE | F | t | p | R square |
| --- | --- | --- | --- | --- | --- | --- |
|  |  |  |  |  |  |  |
| Model predicting neglect |  |  | 15.86 |  | < .001** | .34 |
| Desirability | -.08 | .01 |  | -2.2 | .02* |  |
| MBI | -.11 | .01 |  | -.59 | .57 |  |
| PBA | -.06 | .01 |  | -.23 | .87 |  |
| PHQ-9 | -.40 | .03 |  | -1.39 | .19 |  |
| GAD-7 | .08 | .03 |  | .28 | .76 |  |
| Narcissism | -.04 | .17 |  | -.30 | .79 |  |
| Psychopathy | .34 | .21 |  | 1.86 | .15 |  |
| Machiavellianism | -.50 | .17 |  | -2.9 | .03* |  |
| ASP | .05 | .18 |  | .33 | .74 |  |
| BCAP | -.34 | .49 |  | -1.9 | .14 |  |
| BEST | 1.09 | .03 |  | 5.0 | .01* |  |
| MBI*Age Parent | .17 | .001 |  | .90 | .39 |  |
| PBA*Age Parent | .32 | .001 |  | 1.36 | .38 |  |
| PHQ-9*Age Parent | .45 | .001 |  | 1.57 | .13 |  |
| GAD-7*Age Parent | -.16 | .001 |  | -.60 | .50 |  |
| Narcissism*Age Parent | -.05 | .005 |  | -.19 | .87 |  |
| Psychopathy*Age Parent | -.46 | .005 |  | -2.05 | .12 |  |
| Machiavellianism*Age Parent | .73 | .004 |  | 3.26 | .01* |  |
| ASP*Age Parent | -.04 | .005 |  | -.19 | .85 |  |
| BCAP*Age Parent | .24 | .01 |  | .94 | .47 |  |
| BEST*Age Parent | -.70 | .001 |  | -3.1 | .11 |  |

*Note.* N = 680, excluding cases listwise*.* Desirability = Social Desirability; MBI = Maslach Burnout Inventory General Survey; PBA = Parental Burnout Assessment; PHQ-9 = Patient Health Questionnaire; GAD-7 = Generalized Anxiety Disorder; ASP = Assessment of Sadistic Personality; BCAP = Brief Child Abuse Potential; BEST = Borderline Evaluation of Severity over Time. Variables that are “variable name*Age Parent” consist of interaction term to test the moderating effect of the age of the parent. Bootstrap of 1000 samples.

**Supplementary material 8**

*Hierarchical regression analysis: Variables predicting violence, with desirability controlled, and age of parent as a moderator*

| Effect | Standardized Estimates | SE | F | t | p | R square |
| --- | --- | --- | --- | --- | --- | --- |
|  |  |  |  |  |  |  |
| Model predicting violence |  |  | 28.40 |  | < .001** | .48 |
| Desirability | -.02 | .01 |  | -.67 | .49 |  |
| MBI | -.26 | .01 |  | -1.58 | .14 |  |
| PBA | .25 | .01 |  | 1.12 | .42 |  |
| PHQ-9 | -.03 | .03 |  | -.13 | .89 |  |
| GAD-7 | -.30 | .02 |  | -1.26 | .22 |  |
| Narcissism | .11 | .12 |  | .81 | .40 |  |
| Psychopathy | .15 | .17 |  | .91 | .44 |  |
| Machiavellianism | -.37 | .17 |  | -2.5 | .12 |  |
| ASP | .13 | .19 |  | .92 | .47 |  |
| BCAP | -.48 | .39 |  | -3.07 | .02* |  |
| BEST | 1.68 | .02 |  | 8.73 | <.001** |  |
| MBI*Age Parent | .21 | .001 |  | 1.21 | .22 |  |
| PBA*Age Parent | .10 | .001 |  | .47 | .71 |  |
| PHQ-9*Age Parent | -.02 | .001 |  | -.08 | .93 |  |
| GAD-7*Age Parent | .28 | .001 |  | 1.18 | .23 |  |
| Narcissism*Age Parent | -.19 | .003 |  | -.88 | .34 |  |
| Psychopathy*Age Parent | -.16 | .004 |  | -.81 | .46 |  |
| Machiavellianism*Age Parent | .56 | .004 |  | 2.81 | .07 |  |
| ASP*Age Parent | -.07 | .005 |  | -.38 | .76 |  |
| BCAP*Age Parent | .68 | .01 |  | 2.95 | .02* |  |
| BEST*Age Parent | -1.35 | .001 |  | -6.96 | .01* |  |

*Note.* N = 680, excluding cases listwise*.* Desirability = Social Desirability; MBI = Maslach Burnout Inventory General Survey; PBA = Parental Burnout Assessment; PHQ-9 = Patient Health Questionnaire; GAD-7 = Generalized Anxiety Disorder; ASP = Assessment of Sadistic Personality; BCAP = Brief Child Abuse Potential; BEST = Borderline Evaluation of Severity over Time. Variables that are “variable name*Age Parent” consist of interaction term to test the moderating effect of the age of the parent. Bootstrap of 1000 samples.
